# Atypical Presentation of Post-sternotomy Sternal Osteomyelitis: The Role of Multimodal Imaging in Community Hospital Diagnosis

**DOI:** 10.7759/cureus.117875

**Published:** 2026-10-13

**Authors:** Marcos Rosa Santana, Udesh Pandey, Navya Mandalapu, Ingrid Penate

**Affiliations:** 1 Internal Medicine, BronxCare Health Systems, New York, USA; 2 Medicine, BronxCare Health Systems, New York, USA

**Keywords:** antibiotic treatment, conservative treatment, deep sternal wound infection, osteomyelitis, post-sternotomy infection, post-surgical complication, sternum infection

## Abstract

Sternal osteomyelitis is an uncommon but serious complication of median sternotomy and may present with atypical manifestations that delay diagnosis. It is considered a form of deep sternal wound infection (DSWI) that extends beyond the deep fascia and may involve the sternal bone and adjacent mediastinal tissues. We report a case of delayed post-sternotomy sternal osteomyelitis presenting four months after coronary artery bypass grafting.

A 70-year-old woman with obesity and poorly controlled diabetes mellitus presented with atypical chest pain and a localized mid-sternal rash first noticed less than one week prior. Acute coronary syndrome was ruled out on admission. Laboratory evaluation was significant for markedly elevated inflammatory markers (CRP 134.1 mg/L, ESR 101 mm/hr). Contrast-enhanced computed tomography of the chest and bone scintigraphy revealed supportive findings consistent with active sternal infection.

The diagnosis of sternal osteomyelitis was made based on a compatible clinical presentation following prior sternotomy, markedly elevated inflammatory markers, and concordant multimodal imaging evidence of sternal involvement. Given the absence of hemodynamic instability, mediastinal extension, sternal instability, or purulent discharge, the patient was managed medically with intravenous, followed by oral, antimicrobial therapy. Her clinical status and laboratory findings improved, and surgical debridement was not required.

This case highlights an atypical presentation of delayed post-sternotomy sternal osteomyelitis and the diagnostic value of multimodal imaging in community hospital settings when tissue confirmation is deferred. It further supports conservative medical management with close monitoring in selected patients without features of mediastinitis or structural compromise.

## Introduction

Sternal osteomyelitis is an uncommon but serious complication following median sternotomy and may present with atypical manifestations that delay diagnosis. It is considered a form of deep sternal wound infection (DSWI), described as a postoperative infection that extends beyond the deep fascia and may involve the sternal bone with or without adjacent mediastinal extension [1,2]. DSWI is relatively infrequent, with a reported incidence of 0.3% to 8%, and established risk factors include obesity, smoking, and diabetes mellitus [3]. It is associated with substantial morbidity and prolonged recovery [1,2].

Delayed presentations pose additional diagnostic challenges, particularly when classic features such as wound dehiscence, purulent drainage, or systemic toxicity are absent. In such cases, nonspecific symptoms may predominate, increasing the risk of missed or late recognition. Early clinical suspicion, supported by appropriate laboratory and imaging evaluation, is therefore essential in high-risk patients with prior sternotomy [3].

We report a case of delayed post-sternotomy sternal osteomyelitis presenting four months after cardiac surgery with a thoracic rash and atypical chest pain in a patient with obesity and poorly controlled diabetes mellitus. This case highlights the diagnostic value of multimodal imaging in a community hospital setting and emphasizes the importance of timely recognition to enable appropriate management and prevent progression of this potentially serious postoperative complication.

## Case presentation

A 70-year-old woman presented to the emergency department with one week of pressure-like chest pain and three days of a mid-sternal rash. Her medical history was significant for hypertension, hyperlipidemia, poorly controlled diabetes mellitus (HbA1c 12.5%), chronic anemia, heart failure with reduced ejection fraction, and severe multivessel coronary artery disease status post coronary artery bypass grafting via median sternotomy four months earlier. She denied tobacco use and chronic alcohol consumption. Her body mass index was 32.1 kg/m².

Chest pain was non-radiating, rated 5/10 in intensity, and began while carrying heavy groceries. It worsened with exertion and was reproducible on chest wall palpation. She denied fever, chills, dyspnea, palpitations, syncope, or recent trauma.

On presentation, temperature was 98.3°F, heart rate 64 beats/min, blood pressure 134/67 mmHg, respiratory rate 20 breaths/min, and oxygen saturation 96% on room air. She appeared comfortable and in no acute distress. Cardiovascular examination revealed normal heart sounds without murmurs. Lung fields were clear to auscultation bilaterally. Examination of the sternotomy site showed a well-healed midline scar with an erythematous macular rash that was non-pruritic and non-painful. There was no warmth, fluctuance, drainage, wound dehiscence, crepitus, or sternal instability.

The electrocardiogram showed normal sinus rhythm with right bundle branch block (RBBB). Initial evaluation for acute coronary syndrome was unrevealing, with negative troponin (Troponin T 10 ng/L, normal ≤12 ng/L) and chest radiography showing no acute cardiopulmonary abnormality.

Pertinent laboratory studies obtained on the day of admission are listed in Table 1. Inflammatory markers were markedly elevated, with C-reactive protein of 134.10 mg/L and erythrocyte sedimentation rate of 101 mm/hr.

**Table 1:** Laboratory studies on admission.

Parameter	Value	Reference Range
Hemoglobin	11.6 g/dL	12.0–16.0 g/dL
White blood cell count	8.9 k/µL	4.8–10.8 k/µL
Platelet count	483 k/µL	150–400 k/µL
Sodium	134 mEq/L	135–145 mEq/L
Potassium	4.0 mEq/L	3.5–5.0 mEq/L
Chloride	92 mEq/L	98–108 mEq/L
Bicarbonate	28 mEq/L	24–30 mEq/L
Blood urea nitrogen	32 mg/dL	6.0–20.0 mg/dL
Creatinine	1.0 mg/dL	0.5–1.5 mg/dL
Glucose	322 mg/dL	70–120 mg/dL
Pro-B-type natriuretic peptide	1447 pg/mL	0–125 pg/mL
D-dimer	1168 ng/mL	0–230 ng/mL
Ferritin	270.0 ng/mL	13.0–150.0 ng/mL
Lactate dehydrogenase	151 U/L	110–210 U/L
HbA1c	12.5%	4.7–6.4%
C-reactive protein	134.10 mg/L	≤5.00 mg/L
Erythrocyte sedimentation rate	101 mm/hr	≤30 mm/hr

An elevated D-dimer level prompted computed tomography (CT) of the chest using a pulmonary embolism protocol, which showed no pulmonary embolism. The markedly elevated D-dimer was attributed to the systemic inflammatory process rather than thromboembolic disease, consistent with the subsequent imaging findings.

Additionally, a CT chest finding showed postsurgical changes related to prior median sternotomy, including adjacent subcutaneous edema, punctate foci of subcutaneous air at the incision site, and bony resorption at the sternotomy site (Figure 1). Additionally, borderline mediastinal lymphadenopathy was noted, including an 11-mm short-axis left superior mediastinal lymph node abutting the aortic arch and a 10-mm paratracheal lymph node. The radiology report indicated that while such findings can be seen after recent surgery, the interval of approximately four months since sternotomy made routine postsurgical changes less likely, and the findings were concerning for osteomyelitis. Notably, no drainable abscess, retrosternal fluid collection, or mediastinal inflammatory changes were identified.

**Figure 1:**
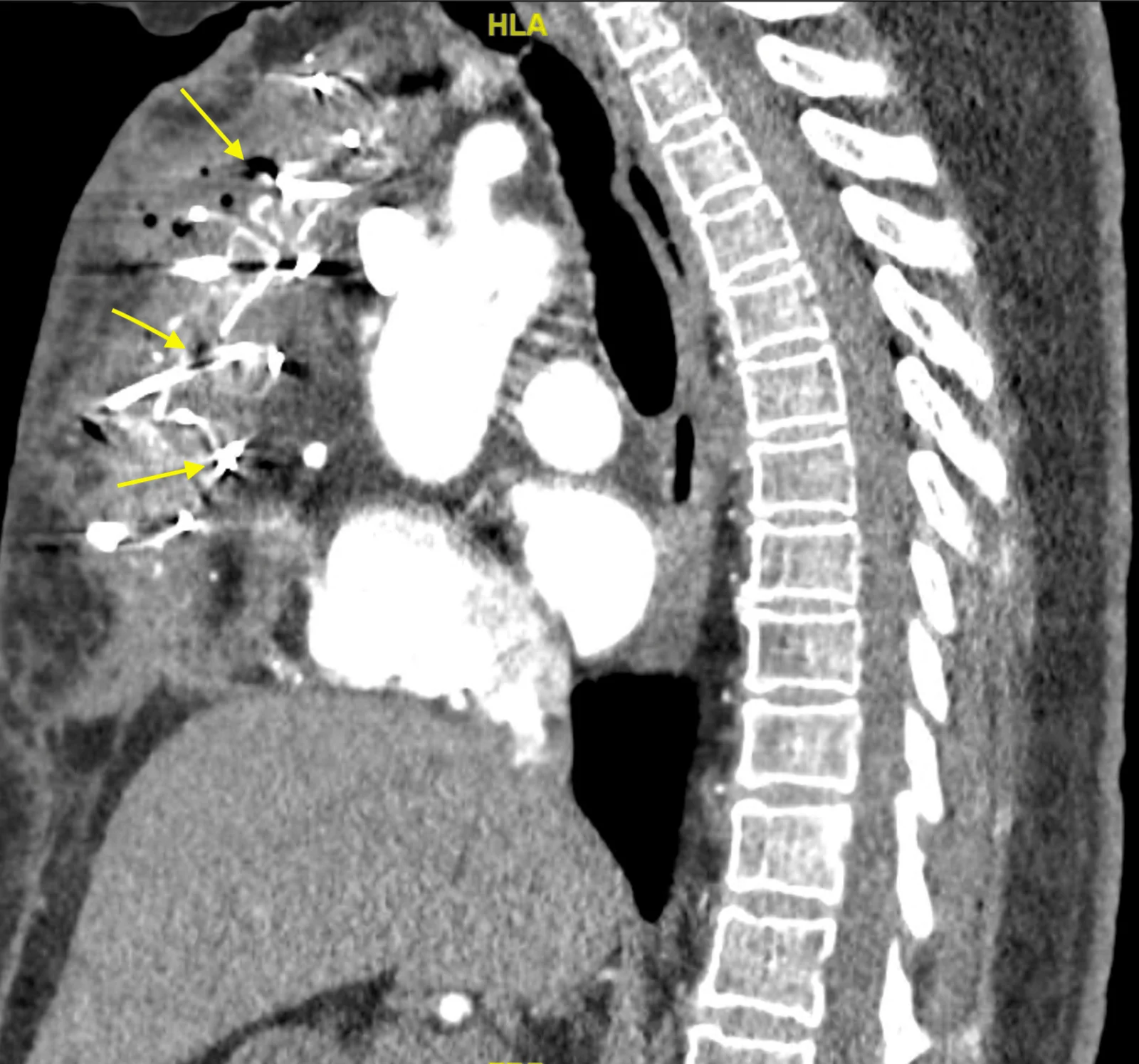
Sagittal contrast-enhanced CT demonstrating subcutaneous edema, punctate foci of subcutaneous air, and bony resorption at the sternotomy site. No retrosternal collection or mediastinal extension was identified.

To further evaluate for suspected sternal infection, a three-phase bone scintigraphy study of the chest was performed with intravenous administration of Tc-99m MDP on hospital day three. It demonstrated persistent delayed-phase radiotracer uptake involving the sternum (Figure 2). These findings were interpreted as consistent with sternal osteomyelitis in the context of the clinical presentation and CT abnormalities, although persistent uptake from surgical remodeling could not be entirely excluded.

**Figure 2:**
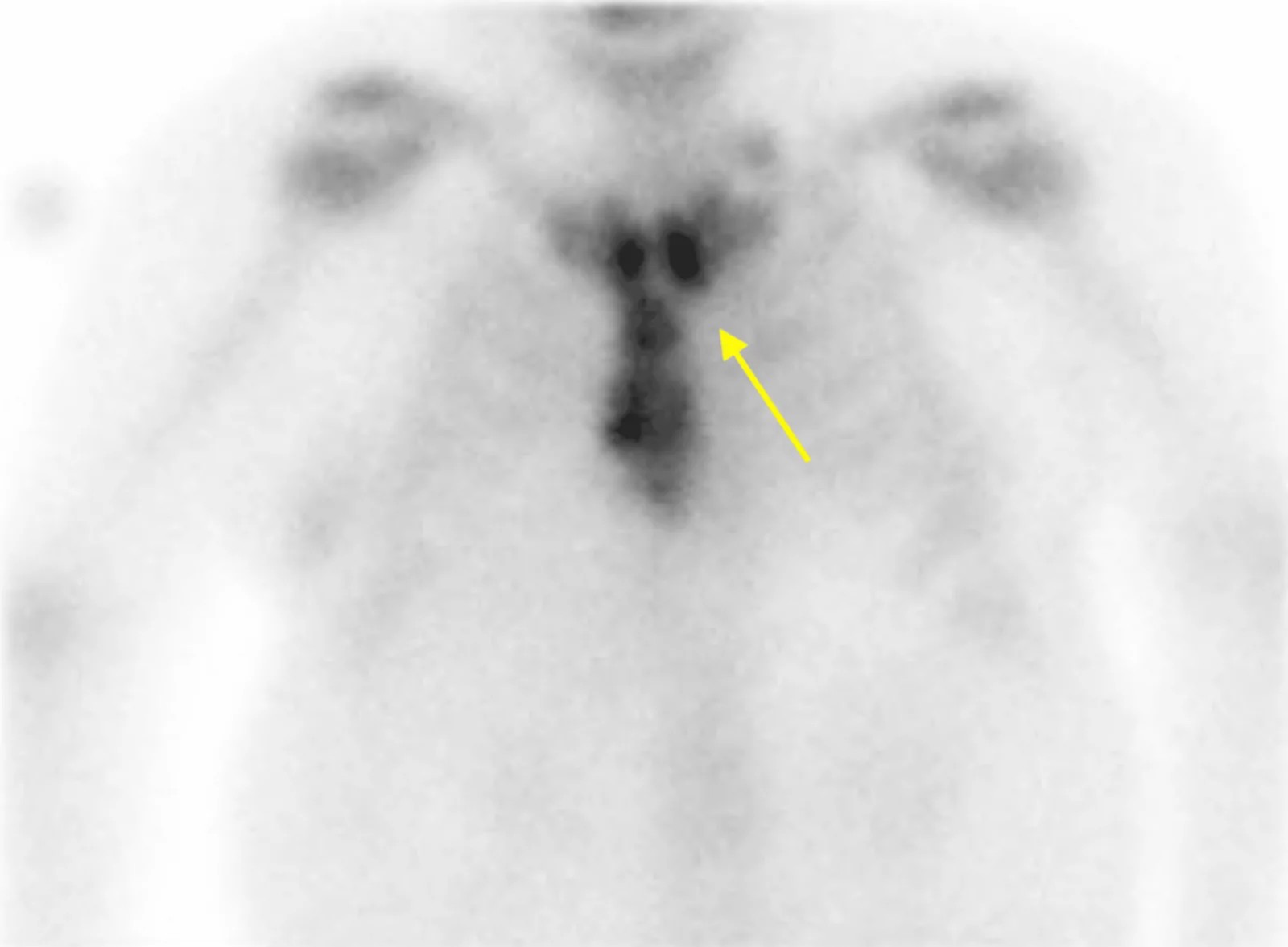
Three-phase Tc-99m MDP bone scintigraphy, delayed phase. Persistent delayed-phase radiotracer uptake involving the sternum, consistent with osteomyelitis.

A diagnosis of probable post-sternotomy sternal osteomyelitis was made based on the compatible clinical features, markedly elevated inflammatory markers, and concordant multimodal imaging findings. Important differential diagnoses included superficial cellulitis and non-infectious postoperative changes. Mediastinitis and abscess were considered less likely based on clinical stability and imaging.

Empiric intravenous vancomycin (1 g every 12 hours) and piperacillin-tazobactam (3.375 g every 6 hours) were initiated on hospital day three. Blood cultures collected on the same day showed no growth. The patient remained hemodynamically stable without systemic toxicity. She initially requested transfer to the surgical center where her coronary artery bypass grafting had been performed. The operating cardiothoracic surgeon was contacted and did not recommend surgical debridement, considering the imaging findings insufficient to warrant operative intervention. Infectious disease consultation favored nonoperative management given the absence of mediastinitis, drainable collections, or structural compromise, and recommended continuation of intravenous antibiotics during hospitalization.

Given clinical stability and the absence of operative indications at the time of discharge, oral trimethoprim-sulfamethoxazole (800 mg/160 mg every 12 hours) was selected as the step-down oral agent, with a planned total antimicrobial course of six to eight weeks. While trimethoprim-sulfamethoxazole is not specifically recommended in published guidelines for sternal osteomyelitis, it was considered a pragmatic option in the context of culture-negative disease, clinical improvement on broad-spectrum intravenous therapy, and close outpatient follow-up arranged with cardiothoracic surgery and infectious disease.

During hospitalization, chest pain resolved, and the rash showed partial improvement. Inflammatory markers trended downward (Table 2).

**Table 2:** Trend of inflammatory markers during hospitalization.

Parameter	Day of Admission	Day 3 of Admission	Reference Range
C-reactive protein (CRP)	134.10 mg/L	40.43 mg/L	≤5.00 mg/L
Erythrocyte sedimentation rate (ESR)	101.0 mm/hr	64.0 mm/hr	≤30 mm/hr

No procedural interventions were performed. The patient was discharged in stable condition with outpatient follow-up arranged with cardiology, infectious disease, and her cardiothoracic surgeon.

At four months' follow-up, she reported no symptoms, examination showed resolution of the rash, and no surgical intervention had been required. Her functional status was unchanged compared to baseline.

## Discussion

Sternal osteomyelitis represents an entity within the spectrum of DSWI, a potentially serious complication following median sternotomy. DSWI is broadly defined as a postoperative infection extending beyond the deep fascia that may involve the sternum, retrosternal tissues, and mediastinal structures [1,3,4].

The reported incidence of DSWI varies across institutions and populations, with published rates ranging from approximately 0.3% to 8% [1,5]. Although relatively uncommon, DSWI carries substantial morbidity, prolonged hospitalization, increased healthcare costs, and increased mortality. In a large population-based cohort, one-year mortality was 10.7% in patients with DSWI, compared to 2.5% in those without [6]. Vigilance, early recognition, and timely intervention are therefore critical in preventing disease progression and further complications, particularly in high-risk populations.

Risk factors for DSWI include both patient-related and procedure-related variables. Patient-related risk factors include advanced age, obesity, diabetes mellitus, chronic lung disease, and smoking. Surgical factors such as prolonged operative time and early revision procedures may further predispose to infection, partly through conditions such as hematoma formation that favor bacterial proliferation. The patient described had poorly controlled diabetes and obesity with a body mass index of 32.1 kg/m², both of which are established risk factors associated with DSWI [1,3].

The clinical presentation of DSWI and sternal osteomyelitis is heterogeneous. Classic findings include wound dehiscence, local tenderness, erythema, purulent drainage, fever, and sternal instability. However, delayed presentations months after surgery are well described and may manifest with subtle or atypical features such as persistent pain or poor wound healing, which can make recognition difficult [2,7]. Our case is notable for its delayed onset several months after surgery, minimal local findings, and the absence of systemic features, all of which can obscure timely recognition.

Historically, several frameworks have been used to classify DSWI. The Pairolero and Arnold classification (1986) is based on the timing and nature of clinical findings. Type I infections present within days of sternotomy with wound separation, with or without sternal instability, and without mediastinal suppuration. Type II infections, the most common form, occur within weeks and typically involve cellulitis, purulent drainage, and communication with the sternum and mediastinum; osteomyelitis is common. Type III infections develop months after surgery and are characterized by a chronic draining sinus tract extending to the sternum or costochondral junctions [8].

In our case, within the Pairolero and Arnold framework, the delayed timing and presence of osteomyelitis most closely resemble Type III disease; however, the absence of a chronic draining sinus tract means that the case does not fully conform to the classic Type III phenotype. It is also inconsistent with Type II disease, given the lack of early postoperative onset, purulent drainage, or mediastinal suppuration.

El Oakley and Wright proposed an alternative classification system that distinguishes five types of DSWI based on the timing of onset, presence of risk factors, and prior treatment attempts, encompassing a spectrum from early uncomplicated infections to late recurrent disease following failed surgical repair [9]. Our case shares features consistent with a delayed, clinically stable presentation involving isolated sternal involvement without mediastinal extension. These observations illustrate the limitations of existing taxonomies in atypical, imaging-defined cases recognized before the development of advanced local complications.

Diagnostic approaches focus on microbiological confirmation, surgical findings, or compatible clinical features supported by objective evidence of infection. The Centers for Disease Control and Prevention (CDC) criteria for post-sternotomy mediastinitis require at least one of the following: (I) an organism isolated from culture of mediastinal tissue or fluid; (II) evidence of mediastinitis seen during surgery; or (III) the presence of chest pain, sternal instability, or fever (>38°C) in combination with either purulent discharge from the mediastinum or isolation of an organism from culture of blood or mediastinal drainage [10]. These criteria were not met in our patient, consistent with the clinical and imaging findings that excluded mediastinal involvement.

In our case, the diagnosis was most consistent with post-sternotomy sternal osteomyelitis based on a history of prior sternotomy, localized symptoms, markedly elevated inflammatory markers, and supportive multimodal imaging findings. Microbiologic confirmation was deferred given clinical stability, absence of drainable collections, and lack of operative indications.

Laboratory testing provides supportive evidence but does not independently establish the diagnosis. Elevated white blood cell count, CRP, and ESR are commonly used markers [1,5]; however, they lack specificity and sensitivity in the postoperative setting. Markedly elevated inflammatory markers therefore warrant further evaluation for deeper infection [1]. In this patient, CRP and ESR were substantially elevated four months after sternotomy, strengthening suspicion for a pathological inflammatory process rather than residual postoperative changes.

Blood cultures are recommended because bacteremia is frequently observed in postoperative mediastinitis, with reported rates ranging from 18% to 57% [1]. However, negative cultures do not exclude infection, as microbiological yield may be limited, particularly in patients previously exposed to antibiotics or in localized infections [7].

Imaging plays a central role in the diagnosis and anatomical assessment of DSWI. Chest radiography may demonstrate mediastinal widening but cannot reliably define the extent of infection [1]. CT, owing to its wide availability, has emerged as a key imaging modality. A systematic review demonstrated CT to be a valuable, noninvasive tool for diagnosing sternal wound infection and delineating the anatomical components of disease, including sternal osteomyelitis and mediastinitis. Reported CT findings associated with infection include free gas, pleural effusions, mediastinal soft-tissue abnormalities, capsular ring enhancement, fluid collections, abscess formation, and sternal erosions or dehiscence [1,5].

A systematic analysis of 25 studies evaluating the utility of CT scanning for sternal wound infections reported that diagnostic performance varied substantially, with sensitivity ranging from 67% to 100% and specificity from 35.7% to 100% [5]. This variability likely reflects differences in the postoperative timing of imaging and overlap between normal postoperative changes and early infectious findings. Accordingly, CT findings should be interpreted alongside clinical presentation, laboratory markers, and microbiologic or operative evidence rather than in isolation. In our case, CT demonstrated postsurgical changes with adjacent subcutaneous edema, foci of subcutaneous air, and bony resorption at the sternotomy site, findings that were concerning in the context of delayed presentation. Importantly, no drainable collection, retrosternal abscess, or mediastinal extension was identified.

Bone scintigraphy demonstrating persistent delayed radiotracer uptake is considered highly sensitive for osteomyelitis, although its specificity is reduced in the setting of prior surgical intervention [11]. While Tc-99m MDP bone scintigraphy can show increased uptake for months following surgery, persistent delayed-phase sternal uptake at four months post-sternotomy raises concern for underlying pathology, particularly when accompanied by clinical and laboratory abnormalities. In this case, the bone scintigraphy findings were not interpreted in isolation; rather, they were integrated with the CT abnormalities, clinical presentation, and inflammatory marker elevation to support a working diagnosis of probable sternal osteomyelitis.

Magnetic resonance imaging (MRI) can also be a valuable modality to evaluate deep sternal and mediastinal infections. However, its use may be limited in post-sternotomy patients due to practical constraints such as implanted metallic devices, patient tolerance, claustrophobia, prolonged scan time, and limited availability [12]. In our case, MRI was not pursued, as it was unlikely to alter clinical decision-making given the concordant CT and scintigraphy findings.

When possible, microbiologic diagnosis with deep tissue sampling should be obtained to guide targeted antimicrobial therapy. Coagulase-negative staphylococci (CoNS) and *Staphylococcus aureus* are consistently reported as the predominant pathogens in DSWIs [1,2,12]. A retrospective cohort of 70 patients with post-sternotomy chronic osteomyelitis found that Gram-positive bacteria were the most common pathogens, identified in 67% of cases. *Staphylococcus* species accounted for 65%, with CoNS being the predominant organism. Methicillin resistance was observed in 89% of coagulase-negative isolates, and polymicrobial infection was present in 18% of patients [12].

Management of DSWI requires a multidisciplinary approach centered on surgical and antimicrobial strategies. Early and adequate surgical debridement is recommended, with removal of necrotic tissue and infected foreign material when present [1]. Empiric antimicrobial therapy should be initiated once DSWI is suspected. Given that Gram-positive organisms - particularly CoNS and *Staphylococcus aureus* - predominate, empiric coverage is generally directed against these pathogens, with additional tailoring based on institutional resistance patterns and patient-specific risk factors. In settings where methicillin-resistant *Staphylococcus aureus* (MRSA) prevalence is low, broad-spectrum beta-lactam agents such as piperacillin-tazobactam may provide adequate coverage. In contrast, in centers with higher MRSA prevalence, agents active against MRSA, such as vancomycin or daptomycin, may be incorporated into empiric regimens. Therapy should be narrowed when culture results become available. Some authors have suggested prolonged antimicrobial therapy for up to 12 weeks, although this recommendation is largely extrapolated from periprosthetic joint infection literature rather than studies specific to DSWI [1].

However, management should be individualized, and selected cases of chronic post-sternotomy osteomyelitis have been reported to respond to systemic antimicrobial therapy alone when supported by accurate microbiological and radiological evaluation [12]. Selected patients without hemodynamic instability, mediastinitis, abscess formation, or sternal instability may be candidates for conservative treatment following multidisciplinary evaluation.

The present case illustrates a scenario in which nonoperative management was considered appropriate following multidisciplinary evaluation. The patient remained clinically stable, with no mediastinal involvement or drainable collection, and preserved sternal stability. Empiric broad-spectrum intravenous antibiotics were initiated and later transitioned to oral therapy. The patient demonstrated symptomatic improvement, reduction in inflammatory markers, and sustained clinical recovery at four months without the need for surgical intervention.

This clinical course underscores the diagnostic value of multimodal imaging in a community hospital setting, where advanced tissue sampling may not be immediately available or clinically indicated. When CT and bone scintigraphy are interpreted together with clinical and laboratory data, a working diagnosis can be established with sufficient confidence to guide management. Timely recognition of limited disease, as illustrated in this case, may allow targeted antimicrobial therapy and close monitoring before the development of mediastinal involvement or other complications requiring surgical intervention.

Limitations of this report include the absence of microbiologic or histopathologic confirmation, which precludes a definitive diagnosis. The application of Native Vertebral Osteomyelitis (NVO) IDSA guideline duration recommendations to sternal osteomyelitis represents an extrapolation, as evidence specific to this anatomical site remains limited. Furthermore, conclusions regarding the management approach are based on a single case and should therefore be interpreted with caution.

## Conclusions

Delayed post-sternotomy infection may present months after surgery with subtle clinical findings, making early diagnosis challenging. This case demonstrates that sternal osteomyelitis can be recognized at a community hospital through systematic clinical evaluation and concordant multimodal imaging, even when tissue confirmation is not readily available. Although surgical intervention remains the cornerstone of treatment for many cases of DSWI, carefully selected stable patients without mediastinal or structural complications may be successfully managed with conservative antimicrobial therapy following multidisciplinary evaluation. Close outpatient follow-up remains essential to monitor for disease progression or the emergence of indications for surgical intervention.
